# A Mathematical Modeling Approach for Targeted Radionuclide and Chimeric Antigen Receptor T Cell Combination Therapy

**DOI:** 10.3390/cancers13205171

**Published:** 2021-10-15

**Authors:** Vikram Adhikarla, Dennis Awuah, Alexander B. Brummer, Enrico Caserta, Amrita Krishnan, Flavia Pichiorri, Megan Minnix, John E. Shively, Jeffrey Y. C. Wong, Xiuli Wang, Russell C. Rockne

**Affiliations:** 1 Division of Mathematical Oncology, Department of Computational and Quantitative Medicine, Beckman Research Institute, City of Hope National Medical Center, Duarte, CA 91010, USA; abrummer@coh.org; 2 Department of Hematology & Hematopoietic Cell Transplantation, Beckman Research Institute, City of Hope National Medical Center, Duarte, CA 91010, USA; dawuah@coh.org (D.A.); akrishnan@coh.org (A.K.); xiuwang@coh.org (X.W.); 3 Department of Hematologic Malignancies Translational Science, Beckman Research Institute, City of Hope National Medical Center, Duarte, CA 91010, USA; ecaserta@coh.org (E.C.); fpichiorri@coh.org (F.P.); 4 Department of Molecular Imaging and Therapy, City of Hope National Medical Center, Duarte, CA 91010, USA; mminnix@coh.org (M.M.); jshively@coh.org (J.E.S.); 5 Department of Radiation Oncology, City of Hope National Medical Center, Duarte, CA 91010, USA; jwong@coh.org

**Keywords:** CAR-T, targeted radionuclide therapy, TRT, mathematical model, multiple myeloma, immunotherapy, daratumumab, CS1, combination therapy, alpha particle therapy, actinium-225

## Abstract

**Simple Summary:**

Targeted radionuclide therapy (TRT) and immunotherapy, an example being chimeric antigen receptor T cells (CAR-Ts), represent two potent means of eradicating systemic cancers. Although each one as a monotherapy might have a limited effect, the potency can be increased with a combination of the two therapies. The complications involved in the dosing and scheduling of these therapies make the mathematical modeling of these therapies a suitable solution for designing combination treatment approaches. Here, we investigate a mathematical model for TRT and CAR-T cell combination therapies. Through an analysis of the mathematical model, we find that the tumor proliferation rate is the most important factor affecting the scheduling of TRT and CAR-T cell treatments with faster proliferating tumors requiring a shorter interval between the two therapies.

**Abstract:**

Targeted radionuclide therapy (TRT) has recently seen a surge in popularity with the use of radionuclides conjugated to small molecules and antibodies. Similarly, immunotherapy also has shown promising results, an example being chimeric antigen receptor T cell (CAR-T) therapy in hematologic malignancies. Moreover, TRT and CAR-T therapies possess unique features that require special consideration when determining how to dose as well as the timing and sequence of combination treatments including the distribution of the TRT dose in the body, the decay rate of the radionuclide, and the proliferation and persistence of the CAR-T cells. These characteristics complicate the additive or synergistic effects of combination therapies and warrant a mathematical treatment that includes these dynamics in relation to the proliferation and clearance rates of the target tumor cells. Here, we combine two previously published mathematical models to explore the effects of dose, timing, and sequencing of TRT and CAR-T cell-based therapies in a multiple myeloma setting. We find that, for a fixed TRT and CAR-T cell dose, the tumor proliferation rate is the most important parameter in determining the best timing of TRT and CAR-T therapies.

## 1. Introduction

Immunotherapy has now established itself as one of the advanced treatment options in cancer care including metastatic stages, or adjuvant or neoadjuvant settings in many cancers [1]. Immunotherapy attempts to cure cancer by stimulating, arming, and priming the host body’s immune system against the tumor. However, patients on immunotherapy also suffer from immune-related adverse events that limit the dose of the therapeutic agent administered to the patient. Thus, to boost efficacy against cancer tumors, immunotherapy may be combined with other established forms of therapy such as radiation. Several active clinical trials are underway to explore the combination of external beam radiation therapy with immunotherapy [2,3] for an improved survival and toxicity control.

**Figure 1:**
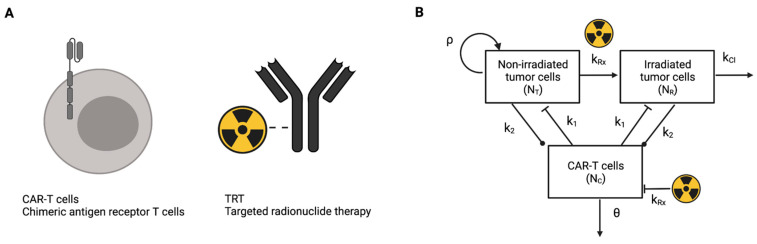
(**A**) Schematic diagram of CAR-T cells and TRT with antibody targeting. (**B**) Diagram of the mathematical model illustrating the relationships and interactions between non-irradiated and irradiated tumor cells and TRT and CAR-T cells.

Chimeric antigen receptor T cell (CAR-T) therapy [4,5,6] is a form of immunotherapy where the T cells are acquired from the patient and are genetically engineered to express a chimeric antigen receptor(s) (CAR) on their surface (Figure 1A). Patient T cells can be engineered to express multiple CARs that can be used to target multiple antigens, which when injected back into the patient target and eradicate the tumor cells based on the antigen expressed. With optimal CAR design and manufacturing, these CAR-T cells further proliferate and survive (persist) in the body, resulting in an increased targeting of the tumor cells. 

Targeted radionuclide therapy (TRT) is a form of radiation therapy where a radionuclide is tagged with another molecule and is injected into the body [7,8]. The affinity of the attached molecule to the tumor cells is exploited to preferentially localize the radiotherapeutic molecule to the target cells. The radionuclide half-life is chosen to best match the kinetics of the molecule targeting the cancer cells; for example, the pharmacokinetic profile of a small molecule or antibody (Figure 1A). Based on the kinetics of the radiotherapeutic and the type and energy deposited by the radionuclide decay, the absorbed dose to the cancer tumor can be determined. The advantage of targeted radionuclide therapy lies in the selectivity of delivering radiation to the cancer cells at the molecular level. Thus, in lieu of external beam radiation therapy, targeted radionuclide therapy is attractive to combine with immunotherapies. In addition, the uptake and thus the absorbed dose to different lesions can be calculated and the therapy planned based on imaging data.

Individually, CAR-T cells or TRT alone may not completely cure the patient of cancer, justifying an effort for a combination of the two therapies. TRT and CAR-T therapies possess unique features that require special consideration when determining how to dose as well as the timing and sequence of combination treatments including the distribution of the TRT dose in the body, the decay rate of the radionuclide, and the proliferation and persistence of the CAR-T cells. These characteristics complicate the additive or synergistic effects of combination therapies and warrant a mathematical treatment that includes these dynamics in relation to the proliferation and clearance rates of the target tumor cells. 

We have previously published mathematical models for the prediction of the tumor response to CAR-T cell therapy in gliomas [9], the chimeric antigen receptor T cell treatment response in gliomas (CARRGO), and for targeted radionuclide therapy [10] in a preclinical multiple myeloma disease model. The purpose of this work is to combine two previously published mathematical models of CAR-T cell and TRT therapies and use the combined and experimentally informed model to explore the impact of dose, timing, and sequencing of these two therapies on tumor growth. 

## 2. Materials and Methods

### 2.1. Mathematical Model

The mathematical models of the tumor response to TRT and CAR-T cell therapies [11] have been described earlier [9,10,12]. The structure of the combined model is illustrated in Figure 1B. 

The mathematical model assumes that the tumor cells are either irradiated ($N_{R}$) or non-irradiated ($N_{T}$) so that the total number of tumor cells is given by $T=N_{T}+N_{R}$ and that CAR-T cells ($N_{C}$) may also be irradiated. CAR-T cells may kill either irradiated or non-irradiated tumor cells at a rate $k_{1}$, may be stimulated to proliferate or to become exhausted upon encounter with a tumor cell at a rate $k_{2}$, and are assumed to die at a rate θ. The rate at which tumor cells or CAR-T cells become irradiated by TRT, given by $k_{Rx}$, is modeled with the linear-quadratic equation with the Lea–Catcheside dose protraction factor [11] to account for the radioactive decay and biological clearance of the radionuclide (λ), tissue repair (γ), and to translate the absorbed radiation dose in units (Gy = J/kg) to a fraction of the cells irradiated. Non-irradiated tumor cells grow exponentially with proliferation rate ρ, which is a net rate of birth minus death rates. Irradiated tumor cells do not proliferate and are cleared out of the system at a rate $k_{cl}$. In this work, we model alpha particle emitting TRT; for example, ^225^Ac-based radionuclides. Thus, the quadratic term in Equation (4) is set to zero (i.e., β=0) to model high linear-energy transfer (LET) alpha particle-based radiation. The initial dose rate is calculated as $R_{0}=\eta A_{0}$ where η is a constant for the conversion of the injected activity $A_{0}$ to the initial dose rate [10]. Mathematically, a treatment is turned on or off with the Heaviside function H(t−τ)**,** which takes a value of zero for t<τ before the start of the treatment and unity for t≥τ during and after treatment. The parameters and values in the model are given in Table 1.

(1) $$\frac{dN_{T}}{dt}=\rho N_{T}-H\left(t-\tau_{TRT}\right)k_{Rx\_T}N_{T}-H\left(t-\tau_{CAR\;T}\right)k_{1}N_{T}N_{C}$$

(2) $$\frac{dN_{R}}{dt}=H\left(t-\tau_{TRT}\right)k_{Rx\_T}N_{T}-H\left(t-\tau_{CAR\;T}\right)k_{1}N_{R}N_{C}-k_{cl}N_{R}$$

(3) $$\frac{dN_{C}}{dt}=k_{2}\left(N_{T}+N_{R}\right)N_{C}-H\left(t-\tau_{TRT}\right)k_{Rx\_C}N_{C}-\theta N_{C}$$

(4) $$k_{Rx\_T}=\alpha_{T}R_{0}e^{-\lambda t}+\frac{2\beta R_{0}^{2}}{\gamma -\lambda }\left(e^{-2\lambda t}-e^{-\left(\lambda +\gamma \right)t}\right)\gamma \lambda$$

### 2.2. Experimental Design and Model Parametrization

The parameters for ^225^Ac-based TRT in the model ($\lambda_{p},\;k_{cl},\;\alpha_{\left\{T,C\right\}},\;\eta$) were derived in our earlier work comparing beta-emitting (^177^Lu-) and alpha-emitting (^225^Ac-) radionuclides in multiple myelomas [10]. The model parameters related to the CAR-T cells ($k_{1},k_{2},\theta$) and the tumor growth rate (ρ) were estimated experimentally with a mouse model of multiple myelomas as follows. Seven mice as a control group were followed using bioluminescence imaging (BLI) and the growth rate (ρ) of the tumor cells was calculated by fitting a monoexponential curve to the data. To obtain a measure of the CAR-T cell death rate θ that was associated with CAR-T cell persistence, three additional mice were intravenously (i.v.) injected with 5 million MM1S multiple myeloma tumor cells that were engineered to express GFP and firefly luciferase [13] and subsequently with 1 million CS1-CAR-T cells (i.v.) on day 7 (Supplemental Figure S1). The BLI images demonstrate the growth and spread of MM1S multiple myeloma cells in the mice (Supplemental Figure S4). Data tables on the experiment are also provided (Supplemental data table SDT1). A more localized measurement of MM cells could be acquired using a PET scan of the mice using ^64^Cu-DOTA-Daratumumab [14]. Once the tumor cells were injected i.v., they were disseminated through the blood into the bone marrow. Based on the BLI, the tumor cells were localized to the bone marrow, first in the larger bones such as the femur, spine, and skull, and then later on to the sternum.

CS1-specific CAR-T cells were generated as previously described [13]. Briefly, leukapheresis products (PBMCs) from healthy donors were depleted of CD14 and CD45RA cells using microbeads. Subsequently, a T naïve/memory population (Tn/mem) characterized by CD62L+ and CD45RO+ cells were enriched from the depleted population using autoMACS. The Tn/mem cells were then activated using CD3/CD28 microbeads and transduced with a second-generation CAR lentivirus consisting of CS1-scFv, an IgG4-hinge region, a 41BB costimulatory domain, and a CD3z signaling domain with a truncated human EGFR domain. Following transduction, the cells were maintained with IL-2 and IL-15 cytokines and expanded for 18–20 days before use. On day 28 following tumor inoculation, the mice were sacrificed, and bone marrow samples were obtained and analyzed using flow cytometry after staining with antibodies against human CD45, CD3, and EGFR (CAR). The number of CAR-T cells and tumor cells in the samples were quantified and the percentage of CAR-T cells compared with the tumor cells (GFP+) was calculated to yield a rough estimate of the parameter θ on day 28. The BLI data reflecting the tumor burden on day 6 and the tumor growth rate (*ρ*) were used to back calculate the tumor burden on day 0 and scale the BLI data to the number of tumor cells (Supplemental Figure S3). The CAR-T cell model parameters $k_{1},k_{2},\;\theta$ were optimized by fitting the model to the average BLI signal of all mice treated with CAR-T cells over time (Supplemental Figure S2). In addition to the BLI data, the estimate of θ on day 28 obtained earlier was used as a data point for optimization. Data table on the experiment are provided (Supplemental data table SDT1).

### 2.3. Mathematical Model Simulations and Analysis

The mathematical model was implemented as follows: 5 million tumor cells were first inoculated in silico at t = 0 and proliferated untreated until day 7, at which point either TRT or CAR-T cell therapy was simulated so that the initial conditions were $N_{T}\left(t=0\right)=5\times 10^{6}$ and $N_{R}\left(t=0\right)=0$. The impact of the therapy was evaluated with three metrics of tumor growth: progression-free survival (PFS), overall survival (OS), and time to nadir, or the minimum tumor burden post-therapy (t_min_). PFS was defined as the last time point where the tumor burden exceeded the tumor burden prior to therapy on day 7. OS was defined as the day the tumor burden reached 10^11^ cells. Four treatment regimens were evaluated with the mathematical model simulations: (1) TRT only; (2) CAR-T cells only; (3) CAR-T cell therapy followed by TRT; and (4) TRT followed by CAR-T cell therapy. The interval between the therapies was varied and the maximum PFS, OS, and t_min_ for these therapeutic regimens as well as the optimal interval between the therapies were investigated. 

To evaluate the sensitivity of the model to the parameters and therapeutic doses, each of these parameters (TRT-injected activity, CAR-T dose, tumor burden, $\rho ,k_{1},k_{2},\theta ,\;\alpha_{c}$) were changed by ±50% and the maximum PFS, OS, and t_min_ were calculated (Supplemental data Tables S1 and S2). The effective decay constant (λ) was changed by only +50% as the physical decay constant of the radionuclide was used in the reference parameter set. A −50% change in λ would not be physiological. Based on this analysis, the most important parameters influencing the outcome were determined. Lastly, PFS and OS were calculated by varying the parameter of highest sensitivity and the implication for optimizing the combination therapy.

## 3. Results

### 3.1. Parameters for the CAR-T Treatment Model

**Figure 2:**
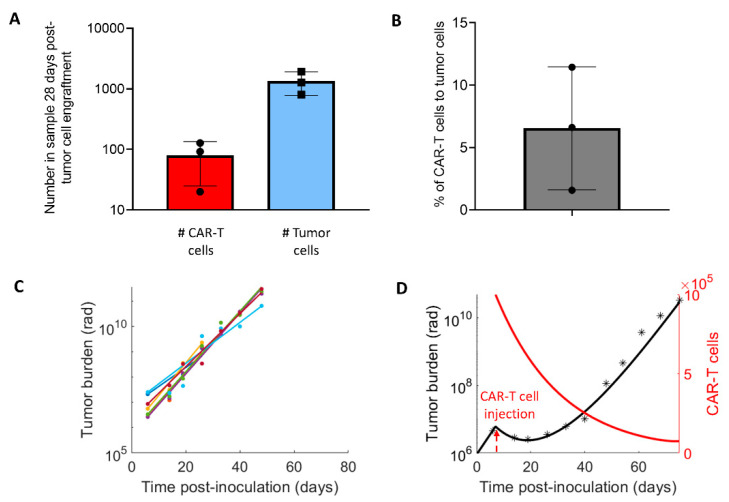
CAR-T cell data and model parameters. (**A**) Estimation of CAR-T cell persistence (θ). Three mice were sacrificed and (**B**) the percentage of CAR-T cells to tumor cells on day 28 post-tumor cell engraftment was used to estimate the CAR-T cell death rate *θ* (1/time). (**C**) Tumor burden as measured with BLI in radiance (rad) for the untreated control mice (N = 7) following the administration of 1 million MM1S multiple myeloma cells at time t=0 to estimate the net tumor growth rate (*ρ*). (**D**) CAR-T cell killing ($k_{1}$) and proliferation/exhaustion ($k_{2}$) parameters are estimated by fitting the mathematical model to the BLI data (*) from mice treated with CAR-T cells on day 7 (N = 3).

Figure 2A shows the number of CAR-T cells and tumor cells as well as the percentage of CAR-T cells (Figure 2B) against the tumor cells obtained from the mice tumor samples on day 28 post-tumor cell engraftment. The percent of CAR-T cells on day 28 to the tumor cells ranged from 1% to 12%. Figure 2C shows the fit of the tumor growth curve to the untreated mice BLI tumor burden data. The simulated tumor burden fitted to the CAR-T mice experiment data (Figure 2D). The CAR-T cell to tumor cell ratio on day 28 found from the fit was 2%.

### 3.2. Evaluating the Therapeutic Regimens

**Figure 3:**
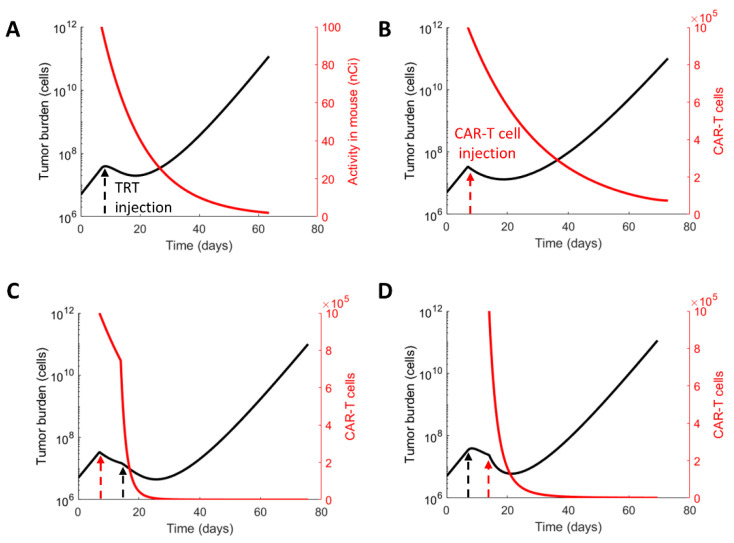
Model simulations of TRT and CAR-T therapy alone and in combination. Monotherapy simulations of (**A**) TRT treatment (black arrow, A_0_ = 100 nCi) or (**B**) CAR-T cell (red arrow, $N_{C}\left(t=7\right)=1\times 10^{6}$) administered on day t=7. Combination treatment simulations with (**C**) CAR-T cells on day t=7 followed by TRT on day 14 and (**D**) TRT on day 7 followed by CAR-T cells on day 14. The mathematical model predicts the tumor growth, radionuclide decay, and CAR-T cell populations over time.

CAR-T cell immunotherapy and targeted radionuclide therapies either as monotherapies or combination therapies were simulated in silico with the mathematical model (Figure 3). A reduced tumor burden was immediately seen post-therapy (day 7) in response to TRT (Figure 3A), or CAR-T therapy (Figure 3B), or a combination of the two therapies when TRT was given 1 week post-CAR-T therapy (Figure 3C), or CAR-T therapy was given 1 week post-TRT (Figure 3D). The sensitivity of the CAR-T cells to TRT resulted in a shorter persistence of CAR-T cells when TRT was given as TRT can kill CAR-T cells (Figure 3D). When a second therapy was given on day 14 as a combination therapy regimen (Figure 3C,D), the model predicted several important effects that were independent of the therapy sequence. Two inflections in the tumor burden curve were evident and the minimum tumor burden in both cases was lower than that obtained by monotherapy alone, showing an additive effect of combination therapy. The time to nadir in the tumor burden also increased along with an increase in progression-free and overall survival (Table 2). The simulations with experimentally derived model parameters (Table 1) showed that the duration of the tumor response (PFS and OS) was prolonged with the CAR-T dose of 1 million cells compared with the TRT-injected activity of 100 nCi. Table 2 shows the time to minimum tumor burden, progression-free survival, or overall survival for each treatment scenario.

### 3.3. Sequence of the Therapy and Maximizing Survival

**Figure 4:**
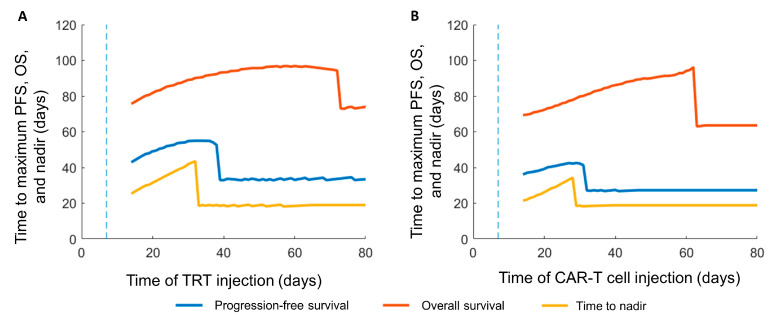
Simulated response to combination TRT and CAR-T cell therapies. Progression-free survival (PFS), overall survival (OS), and time to nadir for two treatment sequences: (**A**) CAR-T cells on day t = 7 (vertical dashed line) followed by TRT starting from t = 14–80. A clear maximum benefit is seen in PFS, OS, and time to nadir. (**B**) TRT on day t = 7 (vertical dashed line) followed by CAR-T starting from t = 14–80. The time to maximum OS, PFS, and nadir is measured from when the tumor is initiated at t = 0.

Figure 4 shows the dependence of progression-free survival, overall survival, and time to tumor burden nadir when comparing the sequence of therapies with either CAR-T cells as the first therapy (Figure 4A) or TRT as the first therapy (Figure 4B). An improvement in both PFS and OS was predicted when CAR-T cells were given prior to TRT. The increased killing of CAR-T cells due to radiation, which was still present post-week 2 due to the long radionuclide half-life, was the reason for the reduced PFS when TRT preceded CAR-T cell therapy. Although a maximum in each of the curves was seen, the curve for overall survival showed that there was a range of timing the second therapy that could yield overall survival very close to the maximum value. The abrupt drop in OS indicated that there was a point in time after which there was no benefit to giving a second therapy regardless of the ordering of the two therapies. The model predicted (Table 2) that, with the experimentally obtained parameters for the current system, CAR-T cell therapy given prior to TRT (Supplemental Video VS1) would increase PFS, OS, and the time to tumor burden nadir compared with TRT given prior to CAR-T cell therapy (Supplemental Video VS2).

### 3.4. The Impact of the Model Parameters and TRT-CAR-T Cell Combination Therapy on Tumor Growth

**Figure 5:**
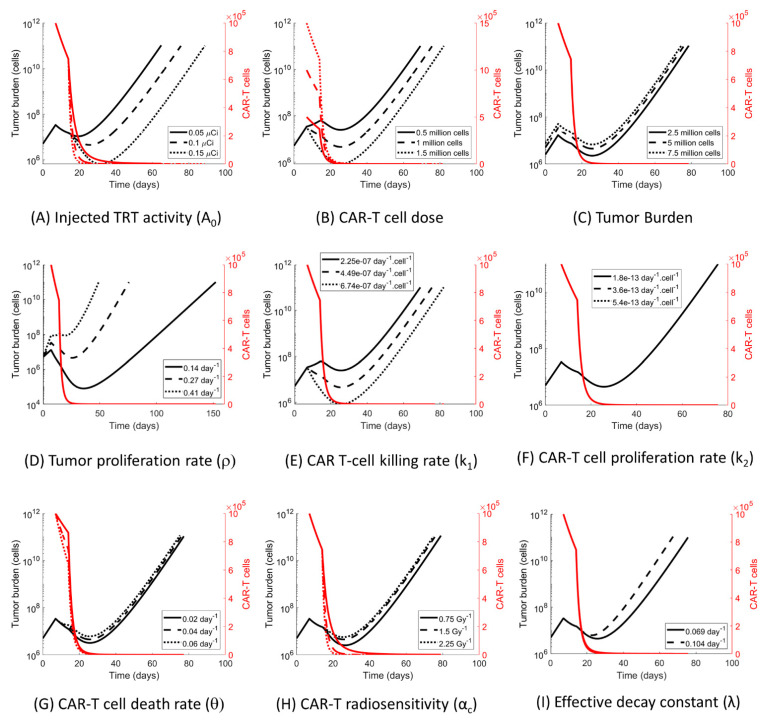
Sensitivity analysis. Impact of the model parameter variation (+/−50%) on the tumor growth curves for the CAR-T (day t = 7) followed by the TRT (day t = 14) therapy for 9 model parameters shown and labeled in Figure subpanels (**A**–**I**). The variation in the tumor proliferation (**D**) has the largest impact on the tumor growth response to combination therapy.

To examine the sensitivity of the model predictions to variations in the parameters, each parameter was changed independently by +/− 50% and a simulation of a combination therapy of CAR-T on day 7 followed by TRT on day 14 was performed (Figure 5). The parameter with the greatest effect on the tumor growth rate was *ρ* whereas the parameter with the least influence was the CAR-T cell proliferation and exhaustion rate *k*_2_. The value of *k*_2_ estimated from the data (Figure 2D) was extremely small and thus its impact on the tumor growth dynamics was also small. In all scenarios, the model predicted that the population of CAR-T cells precipitously dropped following the administration of TRT. Thus, the prediction was that the therapeutic advantage of CAR-T cells in a combination therapy came prior to the administration of TRT due to the effect of radiation on the CAR-T cells. 

**Figure 6:**
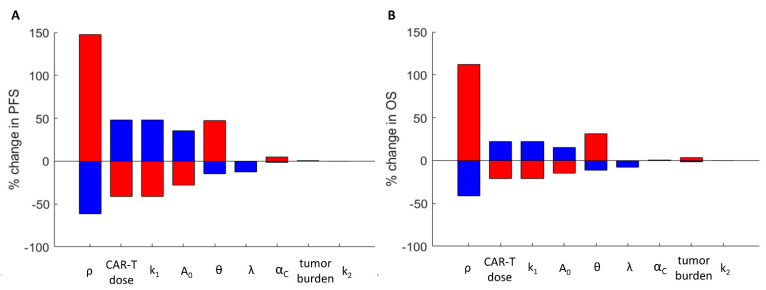
Sensitivity study of the model parameters on the survival outcomes. The change in progression-free survival (**A**) and overall survival (**B**) are shown for a +/−50% change in each of the model parameters. Blue indicates a +50% change and red indicates a −50% change in the parameter. The variations in the tumor proliferation rate have the largest impact on PFS and OS.

Figure 6 summarizes the impact of the model and therapeutic parameters on the predicted PFS and OS. The tumor proliferation rate had the greatest impact on PFS and OS. Using the experimentally derived model parameters, the CAR-T dose was predicted to have a slightly greater impact than TRT on OS and PFS. CAR-T cell radiosensitivity had a greater impact on PFS than OS as the curve for OS was relatively flat over a large range of therapeutic intervals. Conversely, changes in the initial tumor burden impacted OS but did not impact PFS as the tumor dynamics were similar between the two cases and because PFS was a relative measurement from the start of the therapy. The changes in CAR-T cell dose, TRT dose, CAR-T cell killing rate *k*_1_, and proliferation/exhaustion rate *k*_2_ were directly proportional to the changes in PFS and OS; however, an inverse relationship was observed for the tumor proliferation rate *ρ*, CAR-T cell persistence *θ*, effective decay constant λ, tumor burden, and radiation sensitivity of CAR-T cells α_C_. 

**Figure 7:**
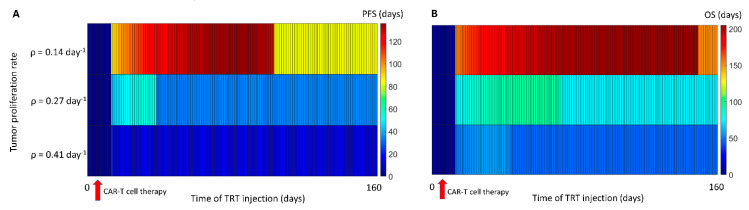
Impact of the tumor proliferation rate on PFS and OS tumor growth. (**A**) PFS. (**B**) OS. Paradoxically, a faster growing tumor results in lower PFS and OS due to a greater response to the treatments. This suggests that the interval between the therapies should be smaller for faster growing tumors.

Based on the results of the sensitivity study, which demonstrated that tumor proliferation was the most important factor influencing PFS and OS, we examined the effect of low, medium, and high tumor proliferation rates on PFS and OS as a function of the time of TRT injection following CAR-T cell therapy (Figure 7). Interestingly, if the tumor growth rate was high, then the PFS and OS were relatively lower due to the increased response to the treatments. However, what was also evident was that the optimal day of administration of the second therapy was also different. The interval between the therapies for tumors that grew faster needed to be reduced compared with a slower growing tumor.

## 4. Discussion

Here, we present a mathematical model combining CAR-T cell immunotherapy and targeted radionuclide therapies for the treatment of cancer with an application to multiple myelomas as an example. The proposed model combined our previously developed models for CAR-T therapy (CARRGO model in [9]) and ^225^Ac-DOTA-daratumumab targeted radionuclide therapy [10]. The model predicted that it is feasible to optimize the dose and timing of the two therapies to maximize tumor growth delay. Using the model parameters derived from the experimental data predicted a better survival outcome when CAR-T cells were given prior to TRT. However, different diseases or therapy combinations might result in different combinations of parameters and potentially different predictions. Thus, it is important to assess the disease or therapy-specific parameters.

A key result of the model was that the time interval between the two therapies should be modified based on the proliferation rate of the tumor. Thus, the measurement of the growth rate of different kinds of cancers can help in the optimization of the combination therapy. Although the model was applied to a setting where the immunotherapeutic was the CS1 CAR-T cell and the radiation therapy was provided by targeted delivery of ^225^Ac-DOTA-Daratumumab to CD38 receptors in multiple myelomas, the model could be applied to general immunotherapeutic and TRT combinations with various targets and therapeutics. Example can be targeting the BCMA CAR-T cells [15,16,17] instead of CS1 CAR-T cells or targeting with a beta particle therapeutic such as ^177^Lu rather than an alpha particle therapeutic such as ^225^Ac.

The mathematical formulation in the proposed model can make assumptions that may be disease- and application-specific. The simplifying assumption of an exponential tumor growth is consistent with the experimental preclinical data presented here; however, the tumor growth rates evaluated at later time points could slow down, reflecting the sigmoidal growth. Clinically, tumors can grow slower than preclinical models where the assumption of an exponential growth rate would suffice. An important aspect to note of the model was the mass-action kinetics of CAR-T cell killing ($k_{1}$) and proliferation/exhaustion ($k_{2}$) that permitted oscillating solutions that were not realistic or likely to be observed in vivo. We noted that, consistent with our prior work in this model [9], the observed parameter ranges did not predict oscillating solutions. 

Additionally, we assumed a monoexponential decay of CAR-T cells; however, there is evidence of a biexponential decay in the CAR-T cell concentration in the blood [18]. A key reason for this assumption is that we used the CAR-T cell percentage measured in the bone marrow rather than in the blood. In this scenario, the magnitude of the exponent of the monoexponential decay would be higher, dominating over a biexponential dynamic. It was assumed in the current work that the CAR-T cells were well-mixed and evenly distributed with the tumor cells. Of course, CAR-T cells can distribute across different organs of the body, potentially increasing the number of CAR-T cells in the tumor sites. The distribution of the CAR-T cells can also be variable across the tumor sites and different CAR-T cell densities can result in a variable response across the tumor sites. Although the well-mixed assumption was reasonable for a disseminated disease such as multiple myelomas, repeated measurements of CAR-T cells in the tumor sites in a preclinical model setting would help support this assumption. In our experimentally derived parameters, the value of *k*_2_ (which indicates the CAR-T cell proliferation or exhaustion) was extremely low compared with the killing rate constant *k*_1_, indicating a very low proliferation of CAR-T cells; thus, the CAR-T cell numbers in the body followed a monoexponential decay curve. However, this might not be the general case. In two lesions evaluated using the CARRGO model [9], the value of *k*_2_ in a lesion with a favorable response to CAR-T cell therapy was significantly higher than a non-responding lesion, indicating a more complex immune system dynamic. 

The model made a simplified assumption that all injected radioactivity (with the same principle applied to CAR-T cells) distributed uniformly through the tumor sites. However, a number of factors hamper this assumption and need to be considered. Radioactivity can be cleared out from the body, taken up non-specifically by normal tissues, or heterogeneously taken up in different tumor locations. If the radioactivity present in the tumor sites was lower than the assumption (as in first two scenarios), the calculated model parameters indicated a lower therapeutic potency of TRT than the actual potency. In the presented model, the tumor burden measurement by BLI resulted in simplifying the application of the model to the data and, at the same time, resulted in the prediction of an average measure for the model parameters and responses. A better measure of the tumor burden and disease localization can be acquired by PET imaging, as has been shown for multiple myelomas both preclinically [14] and clinically [19]. Such an imaging metric can give a spatially variable estimate of the model parameters even at the voxel level. 

^225^Ac is a radionuclide with a multiple progeny that release alpha particles. Although most of them have short enough half-lives to decay at the location of the parent ^225^Ac radionuclide, ^213^Bi has a 45.6 min half-life that can redistribute at a different location from the parent ^225^Ac. Of the four alpha particles released by ^225^Ac and its progeny, one alpha particle is attributed to ^213^Bi. Daratumumab biodistribution is stable in the body over the course of hours–days [19]; this effect was negligible for the model system shown in this manuscript. Apart from the biological redistribution of daughter radionuclides, different radionuclides have different emission particles and ranges that impact on the absorbed fraction of the radiation dose. Although short range alpha particles are absorbed completely locally, therapeutic radionuclides with beta particle emissions have a longer range and might only have a fraction absorbed locally [20]. In this work, we assumed the absorption fraction to be unity but, with more localized imaging data and a different radionuclide, this factor needs to be considered.

A critical issue to consider in combination therapies involving immune and radiation therapies is toxicity. Toxicity in immunotherapy is typically shown in the form of a cytokine release syndrome that limits the dose of the therapeutic administered. Radiation toxicity tends to be proportional to the dose in peripheral organs, mostly in the bone marrow. Apart from individual therapeutic toxicities, the mechanistic interactions between radiation and immunotherapy and the potential common targets of both therapies can pose limits to either of the two therapies and these cells or organs might need to be modeled into the mathematical framework to optimize the dosage, interval, and number of cycles of either of these therapies. There is recent evidence to suggest that radiation can induce changes in the immune system and can stimulate a significant immune response for a better therapeutic efficacy [21,22,23]. Radiation therapy can act as a bridging therapy for immunotherapies yielding better therapeutic efficacies with immunotherapy [24,25,26]. With a better understanding of the mechanistic basis and supporting experimental data, the interactions between radiation and immunotherapy can be better modeled and additional interaction terms can be introduced in the mathematical formulation to account for toxicity. With the current formulation, the effect of radiation resulted in the death of CAR-T cells; thus, it was advantageous to administer CAR-T cells before TRT. However, with the stimulation of the immune system with radiation and the subsequent expansion of the model for radiation-immune interactions, TRT before immunotherapy might present a better therapeutic outcome for survival. The CAR-T cells that are stimulated by radiation can then be separately modeled in the mathematical framework and a result in an increased tumor eradication. 

## 5. Conclusions

With an increasing number of therapies and possible combinations of therapies, it has become essential to incorporate mathematical models to consider the effects of dose, sequence, and timing of multiple therapies. Here we investigated a mathematical model of CAR-T cell immunotherapy and targeted radionuclide therapy. We found that, for a fixed dose of TRT and CAR-T, (1) the tumor proliferation rate was the most important factor in determining the timing between the therapies, and (2) CAR-T cells followed by TRT were more efficacious than TRT followed by CAR-T. These results were specific to the disease model (MM1S multiple myeloma), CAR-T cells (CS1), and TRT (^225^Ac-DOTA-Daratumumab) therapeutic modalities investigated here; however, it is possible that these results may apply to other disease settings. 

## Figures and Tables

**Table 1:** Symbols, values, and references for the parameters in the mathematical model.

Parameter	Symbol	Value	Reference/ Comments
Effective decay constant (1/day)	λ	0.07	Accounts for biological clearance and physical decay
Tumor proliferation rate (1/day)	ρ	0.27	Mean value obtained from untreated controls
Clearance rate of irradiated tumor cells (1/day)	$k_{cl}$	0.5 (Held constant)	[10]
CAR-T cell killing rate (1/day/cell)	$k_{1}$	4.49 × 10^−7^	Optimized from data
CAR-T cell proliferation/ exhaustion rate (1/day/cell)	$k_{2}$	3.6 × 10^−13^	Optimized from data
CAR-T cell death rate (1/day)	θ	0.042	Experimental data and optimization. Range obtained from data is 1–12%
Tumor cell radiosensitivity (1/Gy) *	α_T_	1.5	[10]
CAR-T cell radiosensitivity (1/Gy) *	α_C_	1.5	Assumed equal to tumor radiosensitivity
Activity to dose conversion factor (Gy/day/μCi)	η	3.48	[10]

* Note that the radiosensitivity coefficient incorporates the effect of the radiobiological effectiveness of high linear-energy transfer radiation as is the case in ^225^Ac alpha particle therapy.

**Table 2:** Simulated measures of tumor response to individual and combination therapies. The first therapy is given seven days post tumor initiation. For the combination therapy, the second therapy is given seven days following the first therapy.

Response Criteria	Control	TRT Only (Day 7)	CAR-T Only (Day 7)	CAR-T before TRT	TRT before CAR-T
Progression-free survival (PFS) (days)	-	27	33	55	43
Overall survival (days)	43	64	73	97	96
Time to nadir (days)	-	19	19	44	34
Interval between therapies for maximizing PFS (days)	-	-	-	25	22

## Data Availability

Supplementary data table on the BLI of control and CAR-T cell-treated mice are provided.

## Supplementary Materials

The following are available online at https://www.mdpi.com/article/10.3390/cancers13205171/s1, Figure S1: schematic of CAR-T cell persistence data experiment, Figure S2: BLI images obtained from the CAR-T cell persistence experiment, Figure S3: comparison of temporal development of tumor burden for mice in control group vs. mice treated with CAR-T cell therapy, Figure S4: BLI images obtained from CAR-T cell treatment experiment, Table S1: sensitivity study of model parameters with CAR-T cell therapy prior to TRT, Table S2: sensitivity study of model parameters with TRT prior to CAR-T cell therapy, Video VS1: video showing the simulation of tumor burden with time for CAR-T cell therapy prior to TRT, Video VS2: video showing the simulation of tumor burden with time for TRT prior to CAR-T cell therapy. Supplementary data table SDT1: datasheet with tables on the BLI of control and CAR-T cell-treated mice along with another datasheet showing table on CAR-T cell persistence from tissue studies is provided.
