# Supplementary material for: A Mathematical Modeling Approach for Targeted Radionuclide and Chimeric Antigen Receptor T Cell Combination Therapy
Source: Cancers (Basel). 2021 Oct 15;13(20):5171. doi: 10.3390/cancers13205171 (PMC8533817; doi:10.3390/cancers13205171)
Supplement: Supplementary file 1 [file cancers-13-05171-s001.zip › supplementary/Supplemental_document_TRT_CAR-T_model.docx]

**Sensitivity Study**

Parameters are varied by ± 50% from the values quoted in Table 1 of main text document.

| **Parameter** | **OS** | | | **PFS** | | | **Time to nadir** | | |
| --- | --- | --- | --- | --- | --- | --- | --- | --- | --- |
|  | -50% | 0 | 50% | -50% | 0 | 50% | -50% | 0 | 50% |
| **k_1_** | 77 | 97 | 118 | 32 | 55 | 81 | 28 | 44 | 52 |
| **k_2_** | 97 | 97 | 97 | 55 | 55 | 55 | 44 | 44 | 44 |
| **ρ** | 205 | 97 | 57 | 136 | 55 | 21 | 96 | 44 | 9 |
| **Tumor Burden** | 100 | 97 | 95 | 55 | 55 | 55 | 44 | 44 | 44 |
| **θ** | 127 | 97 | 86 | 81 | 55 | 47 | 61 | 44 | 37 |
| **A_0_** | 83 | 97 | 112 | 39 | 55 | 74 | 26 | 44 | 62 |
| **CAR-T Dose** | 77 | 97 | 118 | 32 | 55 | 81 | 28 | 44 | 52 |
| **α_C_** | 97 | 97 | 97 | 58 | 55 | 54 | 45 | 44 | 42 |
| **λ** | - | 97 | 89 | - | 55 | 48 | - | 44 | 37 |

**Table S1:** Sensitivity of survival outcomes evaluated with respect to the model parameters for CAR-T + TRT therapy. Maximum OS, PFS and time to nadir are calculated by varying the interval between the therapies.

| **Parameter** | **OS** | | | **PFS** | | | **Time to nadir** | | |
| --- | --- | --- | --- | --- | --- | --- | --- | --- | --- |
|  | -50% | 0 | 50% | -50% | 0 | 50% | -50% | 0 | 50% |
| **k_1_** | 75 | 96 | 115 | 34 | 43 | 57 | 26 | 34 | 47 |
| **k_2_** | 92 | 96 | 101 | 43 | 43 | 43 | 34 | 34 | 34 |
| **ρ** | 170 | 96 | 51 | 130 | 43 | 20 | 91 | 34 | 19 |
| **Tumor Burden** | 100 | 96 | 95 | 43 | 43 | 43 | 34 | 34 | 34 |
| **θ** | 126 | 96 | 85 | 46 | 43 | 41 | 36 | 34 | 32 |
| **A_0_** | 83 | 96 | 114 | 24 | 43 | 63 | 21 | 34 | 40 |
| **CAR-T Dose** | 75 | 96 | 115 | 34 | 43 | 57 | 26 | 34 | 47 |
| **α_C_** | 99 | 96 | 94 | 49 | 43 | 39 | 39 | 34 | 32 |
| **λ** | - | 96 | 92 | - | 43 | 35 | - | 34 | 30 |

**Table S2:** Sensitivity of survival outcomes evaluated with respect to the model parameters for TRT + CAR-T therapy. Maximum OS, PFS and time to nadir are calculated by varying the interval between the therapies.

**Figure S1:** Schematic for acquiring CAR-T cell persistence data in mice


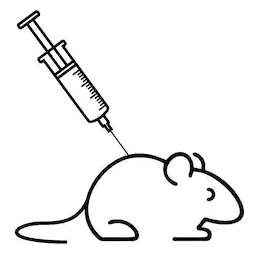


**Engraft**

**5x10^6^ MM.1S eGFP-Ffluc**

**I.V.**

**Day 6**

**Day 0**

**Day 7**

**BLI & Sort**

**CS1CAR-T cell treatment**


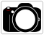

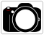


**BLI**

**Day 14**

**Tissue**

**harvest**


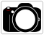


**BLI**

**Day 21**

**Day 28**


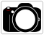


**BLI**


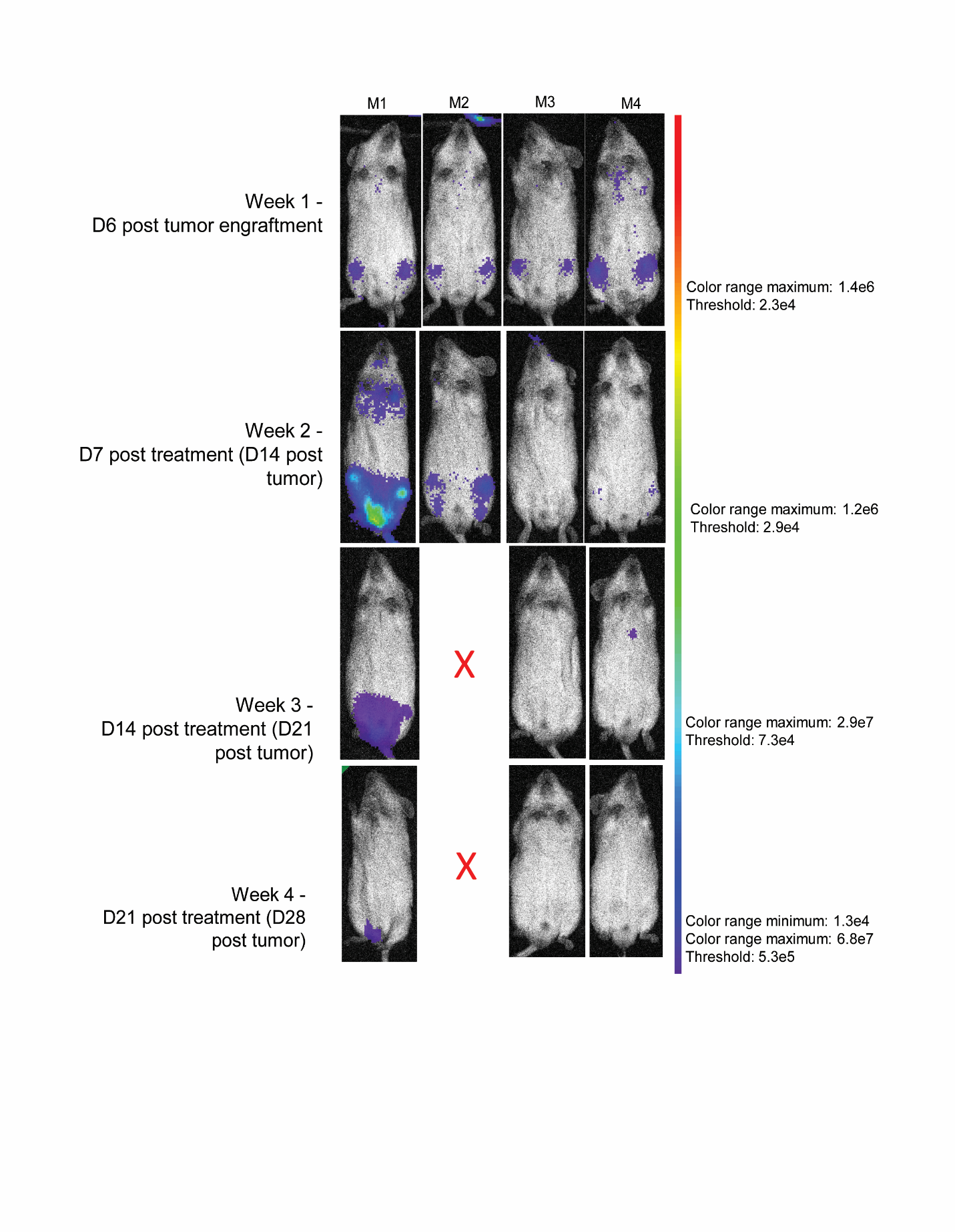


**Figure S2:** Mice BLI images for mice used to acquire CAR-T cell persistence data. BLI images corresponding to CAR-T cell persistence data in Figure 2A and 2B.

**CAR-T cell therapy data used in Figure 2 C,D**

Please see attached Excel sheet for the data used


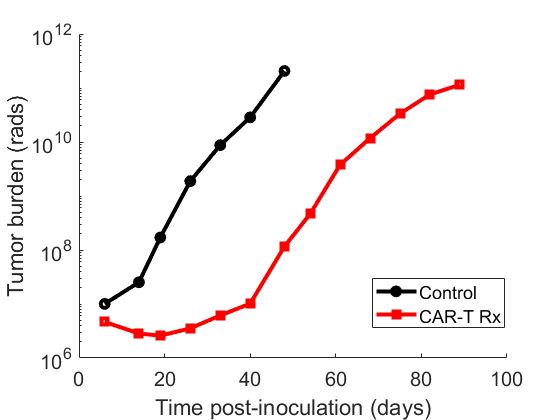


**Figure S3:** Comparison of mean BLI data for control mice vs. mice treated with 100 nCi of ^225^Ac-DOTA-Dara on day 7. Y axis is in the units of photon flux or rads (photons/s/cm^2^).

Figure S3 compares the tumor burden for the control mice against the mice treated with CAR-T cell therapy. Data up to day 26 for the control mice and day 75 for the CAR-T cell treated mice is used.

Bioluminescence images of mice for the control and the CAR-T therapy are shown in figure S4

Accompanying video files shows the changes in tumor growth given different intervals between CAR-T cell + TRT therapy as well as TRT + CAR-T cell therapy


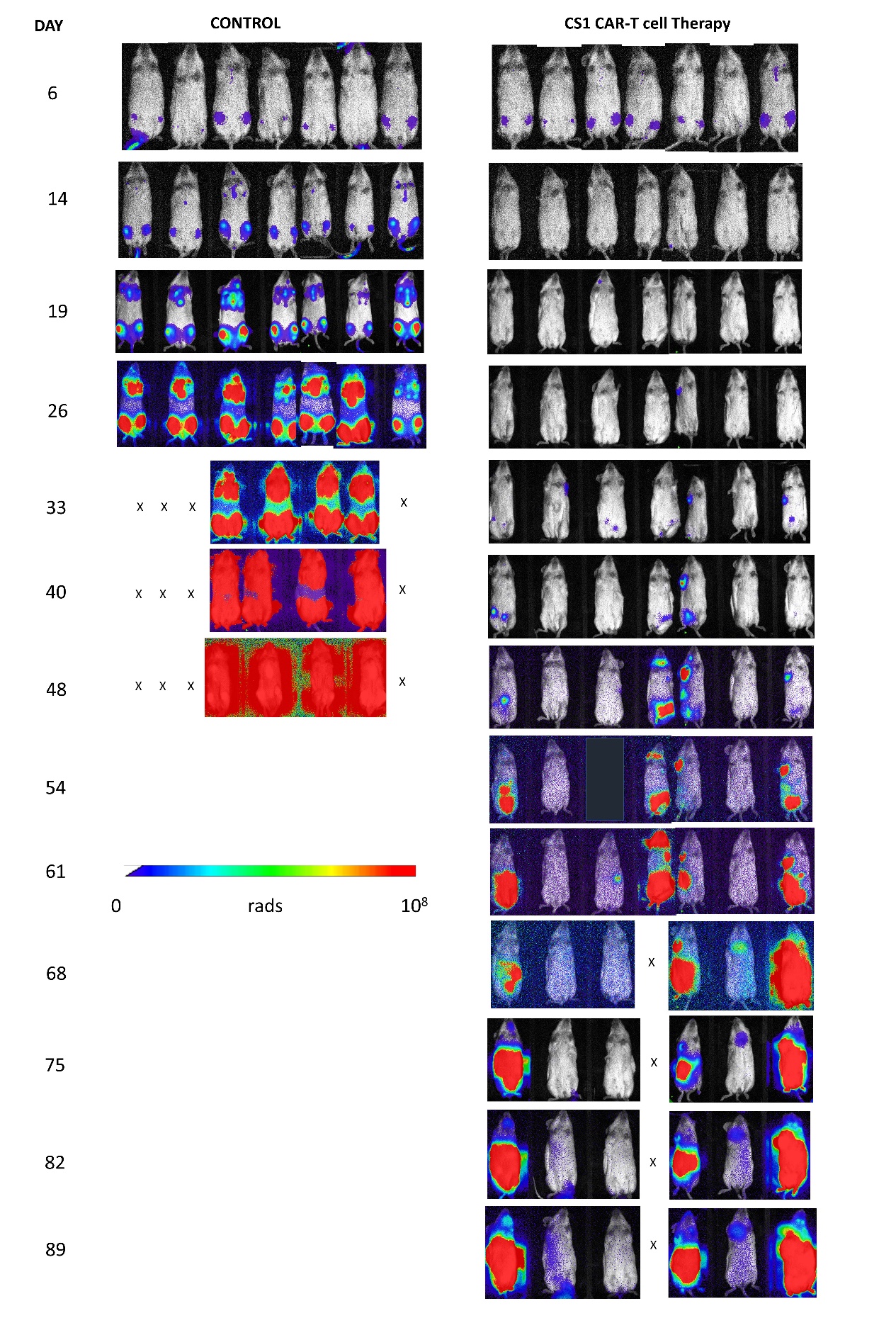


**Figure S4:** Mice BLI images for control and CAR-T cell therapy mice
